# Two new species of the genus *Nephrotoma* (Diptera, Tipuloidea, Tipulidae) from China with a key to species from Mainland China

**DOI:** 10.3897/zookeys.532.5970

**Published:** 2015-11-05

**Authors:** Qiu-Lei Men, Guo-Xi Xue, Hai Yang

**Affiliations:** 1 School of Life Sciences, the Province Key Laboratory of the Biodiversity Study and Ecology Conservation in Southwest Anhui, Anqing Normal University, Anqing, Anhui 246011, P. R. China; 2 School of Food and Bioengineering, Zhengzhou University of Light Industry, No. 5 Dongfeng Road, Zhengzhou, Henan 450002, P. R. China; 3 Administative Bureau of Liankangshan National Nature Reserve, Xin County, Xinyang, Henan 465550, P. R. China

**Keywords:** Nematocera, crane flies, taxonomy, internal reproductive organ, China

## Abstract

*Nephrotoma liankangensis* **sp. n.** and *Nephrotoma pseudoliankangensis* **sp. n.** are described from males and females collected from Henan and Yunnan provinces, China. Morphological descriptions and illustrations for the new species are provided. A key to known species from mainland China is provided. Some internal reproductive structures, including male semen pump, female vaginal apodeme and spermatheca, are described and compared. The possible usefulness of these internal reproductive structures for separating related species is analyzed. The type specimens are deposited in the animal specimen room, School of Life Sciences, Anqing Normal University, Anhui Province, China.

## Introduction

*Nephrotoma* is a large genus in the family Tipulidae. It was originally erected by Meigen (1803) with type species *Tipula dorsalis* (Fabricius, 1781), which is widely distributed in Palaearctic region. This genus is characterized by the following characters: median size; Rs short, cell m1 with or without petiole; ninth tergite covered with small black spines, never completely confused with ninth sternite, has a varied shaped posterior extension; outer gonostylus more or less lobe-shaped, generally flattened, fleshy or partly sclerotized; female cerci longer than hypovalva (Tangelder 1983, 1984). Up to now, 446 species and 29 subspecies of *Nephrotoma* have been reported worldwide, with 78 species recorded from mainland China (Oosterbroek 2015).

During this study of crane fly specimens collected from Henan and Yunnan provinces, China, two new species of *Nephrotoma* were found. Morphological descriptions and detailed illustrations for the new species are provided herein. A key to known species from mainland China is provided. In addition, some internal reproductive structures of the new taxa, including male semen pump, female vaginal apodeme and spermatheca, are described and compared. The possible usefulness of these internal reproductive structures for separating related species is analyzed.

## Material and methods

The specimens examined in this study were collected from Henan and Yunnan provinces by the first author and undergraduates of the School of Life Sciences, Anqing Normal University. The genitalia, including male hypopygium and female ovipositor were removed and soaked in 10% NaOH for 12 hours to clear the muscle for examination. The cleared genitalia were immersed in glycerin jelly, and then examined and drawn using Leica MZ125 (Leica, Germany) stereomicroscope. All measurements were made with the aid of a digital caliper in millimeters (mm). The terminology and methods of description and illustration follow that of Alexander and Byers (1981) and Frommer (1963).

The key was principally constructed from descriptions in the literature without examination of the type species of most of these species, and should be considered preliminary. The characters used in the key rely primarily on the structure of genitalia, the variation of veins and the number of stripes on the prescutum.

### Key to species of the genus *Nephrotoma* from mainland China

**Table:** 

1	Prescutum with stripe(s)	**2**
–	Prescutum without stripe	**3**
2 (1)	Prescutum with only one broad stripe, which almost covers the whole prescutum (see Savchenko 1973: p. 164)	***Nephrotoma villosa* (Savchenko, 1973)** (China: Anhui)
–	Prescutum with more than one stripe	**5**
3 (1)	Stigma covered with macrotrichia	**4**
–	Stigma not covered with macrotrichia (see Yang and Yang 1990: p. 477)	***Nephrotoma hypogyna* Yang & Yang, 1990** (China: Yunnan)
4 (3)	Head and thorax brown with grayish-white pruinosity; wing entirely light brown (see Yang and Yang 1993: p. 54)	***Nephrotoma guangxiensis* Yang & Yang, 1993** (China: Guangxi)
–	Head yellow, thorax brown, without grayish-white pruinosity; wing hyaline, tinged with light brown at apex (see Yang and Yang 1993: p. 56)	***Nephrotoma jinxiuensis* Yang & Yang, 1993** (China: Guangxi)
5 (2)	Prescutum with black stripes	**6**
–	Prescutum with stripes not black	**7**
6 (5)	Prescutal stripes with velvety black margin	**29**
–	Prescutal stripes without velvety black margin	**28**
7 (5)	Prescutum with four stripes	**8**
–	Prescutum with three stripes	**9**
8 (7)	Male tergite nine without median notch, medially terminated into an obtuse lobe (see Yang and Yang 1987b: p. 134, fig. 4B); each flagellomere bicoloured (see Alexander 1925: p. 407)	***Nephrotoma rectispina* Alexander, 1925** (China: Hubei, Guizhou)
–	Male tergite nine with U-shaped notch; flagellum black throughout	**10**
9 (7)	Abdomen without dark apical or lateral stripes	**11**
–	Abdomen with dark apical and/or lateral stripes	**12**
10 (8)	Male tergite nine produced into two lobes; pleura yellow variegated with pale yellow (see Alexander 1949: p. 517)	***Nephrotoma quadrinacrea* Alexander, 1949** (China: Hubei, Guangdong)
–	Male tergite nine produced into four lobes; pleura entirely light yellow (see Alexander 1949: p. 515)	***Nephrotoma progne* Alexander, 1949** (China: Guangdong)
11 (9)	Male tergite nine projected into four lobes	**13**
–	Male tergite nine projected into two lobes	**14**
12 (9)	Occiput with mark	**17**
–	Occiput without mark	**18**
13 (11)	Caudal margin of male sternite eight with an appendage directed caudally (see Yang and Yang 1997: p. 30, Pl. III, fig. 1); abdomen dark brown with two to four segments yellow (see Yang and Yang 1997: p. 30)	***Nephrotoma meridionalis* Yang & Yang, 1997** (China: Hainan, Guangxi)
–	Caudal margin of male sternite eight without appendage; abdomen uniformly coloured	**15**
14 (11)	Cell m1 petiolate (see Alexander 1951: p. 1097); male sternite eight without produced appendage at caudal margin (see Alexander 1967: Pl. IV, fig. 30)	***Nephrotoma inorata* Alexander, 1951** (China: Xizang; India)
–	Cell m1 sessile; male sternite eight with produced appendage at caudal margin	**16**
15 (13)	Occiput without mark (see Yang and Yang 1990: p. 122); male sternite nine without appendage at caudal margin (see Yang and Yang 1990: p. 124, fig. 1A)	***Nephrotoma sichuanensis* Yang & Yang, 1990** (China: Sichuan)
–	Occiput with a linear mark medially; male sternite nine with an appendage bifid and directed caudally (see Tangelder 1984: p. 51, fig. 125; p. 58, fig. 149)	***Nephrotoma koreana* Tangelder, 1984** (China: Hebei, Heilongjiang, Ningxia; Russia; North Korea)
16 (14)	Flagellum dark yellow; scutum with spots brown; abdomen dark yellow (see Yang and Yang 1987: p. 243)	***Nephrotoma qinghaiensis qinghaiensis* Yang & Yang, 1987** (China: Qinghai)
–	Flagellum with first flagellomere yellowish brown, the remainder of flagellum brown; scutum with black spots; abdomen blackish brown (see Yang and Yang 1990: p. 480)	***Nephrotoma qinhaiensis nigrabdomen* Yang & Yang, 1990** (China: Heilongjiang, Inner Mongolia)
17 (12)	Cell m1 sessile	**24**
–	Cell m1 petiolate	**25**
18 (12)	Flagellomeres bicoloured, dark brown on enlarged bases, brown on apical portions of each flagellomere (see Alexander 1914: p. 158)	***Nephrotoma flavonota* (Alexander, 1914)** (China: Zhejiang, Fujian, Hainan; Japan)
–	Flagellomeres uniformly coloured	**19**
19 (18)	Flagellum with first flagellomere yellow, the remainder of flagellum black or brown	**20**
–	Flagellum entirely black	**21**
20 (19)	Inner gonostylus with toothed crest (see Tangelder 1984: p. 85, fig. 277; Yang and Yang 1991: p. 44, fig. 2C)	**22**
–	Inner gonostylus without crest (see Yang and Yang 1990: p. 477, Pl. II, fig. 3)	**23**
21 (19)	Wing weak brown; prescutum with orange stripes (see Alexander 1949: p. 513)	***Nephrotoma citricolor* Alexander, 1949** (China: Fujian)
–	Wing whitish hyaline; prescutum with fuscous stripes (see Matsumura 1916: p. 466)	***Nephrotoma makiella* (Matsumura, 1916)** (China: Fujian, Taiwan)
22 (20)	Male sternite eight without produced appendage at caudal margin (see Tangelder 1984: p. 85, fig. 272); wing yellowish with dark brown stigma (see Alexander 1935: p. 228)	***Nephrotoma profunda* Alexander, 1935** (China: Sichuan, Hubei)
–	Male sternite eight with produced appendage at caudal margin (see Yang and Yang 1991: p. 44, fig. 2A); wing hyaline with light brown stigma	***Nephrotoma hunanensis* Yang & Yang, 1991** (China: Hunan)
23 (20)	Male tergite nine with two horn-shaped processes (see Yang and Yang 1990: p. 477, Pl. II, fig. 2); flagellum with flagellomeres brown except the first yellow	***Nephrotoma ruiliensis* Yang & Yang, 1990** (China: Yunnan)
–	Male tergite nine without horn-shaped processes (Fig. 25); flagellum with flagellomeres black except the first yellow	***Nephrotoma pseudoliankangensis* sp. n.** (China: Yunnan)
24 (17)	Vertex with a triangular stripe; inner gonostylus with a spinous lobe at posterior basal portion (see Alexander 1937: p. 22, fig. 26)	***Nephrotoma stylacantha* Alexander, 1937** (China: Fujian, Jiangsu)
–	Vertex with a thin linear stripe; inner gonostylus without lobe described as above (see Alexander 1941b: Pl. III, fig. 34)	***Nephrotoma alticrista* Alexander, 1941** (China: Sichuan)
25 (17)	Male tergite nine without notch at caudal margin, medially terminated into an obtuse lobe (see Alexander 1940: Pl. VIII, fig. 7)	***Nephrotoma medioproducta* Alexander, 1940** (China: Zhejiang)
–	Male tergite nine with notch in the middle of caudal margin	**26**
26 (25)	Male tergite nine with two spinous processes excluding setae (see Sidorenko 1999: p. 112, Pl. 64, fig. 3)	***Nephrotoma sinensis* (Edwards, 1916)** (China: Beijing, Henan, Jiangsu, Shaanxi, Sichuan, Jiangsu, Yunnan, Hainan, Taiwan; Russia; North Korea; South Korea)
–	Male tergite nine without spinous process excluding setae (see Alexander 1935a: Pl. IV, fig. 50)	**27**
27 (26)	Pleura yellow with a variable amount of red; male tergite nine with a V-shaped notch in the middle of caudal margin (see Alexander 1935a: Pl. IV, fig. 50)	***Nephrotoma omeiana* Alexander, 1935** (China: Sichuan, Hubei, Taiwan)
–	Pleura yellowish brown with a variable amount of white; male tergite nine with a rounded notch in the middle of caudal margin (Fig. 6)	***Nephrotoma liankangensis* sp. n.** (China: Henan)
28 (6)	Scutum uniformly coloured	**30**
–	Scutum with dark stripe or spot, or with different colours in middle portion	**31**
29 (6)	Cell m1 petiolate (see Alexander 1935a: Pl. I, fig. 20; Alexander 1949: p. 519)	**66**
–	Cell m1 sessile (see Alexander 1935c: Pl. I, fig. 15)	***Nephrotoma catenata catenata* Alexander, 1935** (China: Sichuan)
30 (28)	Abdomen not uniformly coloured, with dark stripe on tergites	**32**
–	Abdomen uniformly coloured (see Yang and Yang 1991: p. 39)	***Nephrotoma basiflava* Yang & Yang, 1991** (China: Qinghai)
31 (28)	Occiput without a mark	**34**
–	Occiput with a mark	**35**
32 (30)	Cell m1 sessile (see Alexander 1936a: p. 15)	***Nephrotoma drakanae* Alexander, 1936** (China: Gansu, Inner Mongolia)
–	Cell m1 petiolate	**33**
33 (32)	Occiput without a mark; scutellum orange ochreous (see Edwards 1916: p. 266)	***Nephrotoma parva* (Edwards, 1916)** (China: Jiangxi, Guangxi, Guangdong, Taiwan)
–	Occiput with triangular black spot; scutellum black (see Edwards 1928: p. 700)	***Nephrotoma distans* Edwards, 1928** (China: Xizang)
34 (31)	Antennae black throughout; abdomen chiefly black, with fourth, fifth and base of third tergites orange (see Alexander 1941a: p. 405)	***Nephrotoma aurantiocincta* Alexander, 1941** (China: Sichuan, Yunnan)
–	Antennae variously coloured but not black throughout; abdomen chiefly yellow, with fourth and fifth tergites not orange	**36**
35 (31)	Flagellum with basal three segments yellow (see Yang and Yang 1988: p. 111)	***Nephrotoma catenata guizhouensis* Yang & Yang, 1988** (China: Guizhou)
–	Flagellum with basal segments not yellow, or only first segment yellow	**42**
36 (34)	Abdomen with seventh segment black	**37**
–	Abdomen with seventh segment not black	**38**
37 (36)	Cell m1 long-petiolate, longer than m-m (see Alexander 1936b: Pl. I, fig. 2)	***Nephrotoma hainanica* Alexander, 1936** (China: Hainan)
–	Cell m1 short-petiolate, shorter than m-m (see Alexander 1935b: Pl. I, fig. 4)	***Nephrotoma biarmigera* Alexander, 1935** (China: Zhejiang)
38 (36)	Male sternite eight without produced appendage in the middle of caudal margin (see Yang and Yang 1993: p. 55, fig. 2A; Yang and Yang 1991: p. 44, fig. 1A; Yang and Yang 1990: p. 124, fig, 4A)	**39**
–	Male sternite eight with produced appendage in the middle of caudal margin (see Yang and Yang 1995: p. 420, fig. 1; Yang and Yang 1987b: p. 132, fig. 2A)	**40**
39 (38)	Process of male tergite nine widened basally and narrowed apically (see Yang and Yang 1993: p. 55, fig. 2B)	***Nephrotoma tianlinensis* Yang & Yang, 1993** (China: Guangxi)
–	Process of male tergite nine horn-shaped (see Yang and Yang 1991: p. 44, fig. 1B; Yang and Yang 1990: p. 124, fig. 4B)	**41**
40 (38)	Male sternite nine with an appendage directed ventrally in the middle of caudal margin (see Yang and Yang 1995: p. 420, fig. 1)	***Nephrotoma zhejiangensis* Yang & Yang, 1995** (China: Zhejiang)
–	Male sternite nine without appendage (see Yang and Yang 1987a: p. 132, fig. 2A)	***Nephrotoma geniculata* Yang & Yang, 1987** (China: Inner Mongolia, Hubei, Sichuan, Guizhou)
41 (39)	Abdominal tergites with median stripe; inner gonostylus without crest (see Yang and Yang 1991: p. 44, fig. 1C)	***Nephrotoma cuneata* Yang & Yang, 1991** (China: Hubei, Hunan)
–	Abdominal tergites without median stripe; inner gonostylus with crest (Yang and Yang 1990: p. 124, fig. 4C)	***Nephrotoma xichangensis* Yang & Yang, 1990** (China: Sichuan)
42 (35)	The first flagellomere not the same colour as remaining flagellomeres of flagellum	**43**
–	Flagellum with flagellomeres all the same colour	**44**
43 (42)	Inner gonostylus with crest	**45**
–	nner gonostylus without crest	**46**
44 (42)	Male tergite nine without emargination, or with shallow emargination on caudal margin	**53**
–	Male tergite nine with U-shaped or V-shaped notch on caudal margin	**54**
45 (43)	Crest of inner gonostylus with tooth on dorsal margin	**47**
–	Crest of inner gonostylus without tooth (see Oosterbroek 1985: p. 268, fig. 103)	***Nephrotoma hirsuticauda* Alexander, 1924** (China: Heilongjiang, Inner Mongolia, Gansu, Ningxia; Russia; Japan; Mongolia; North Korea)
46 (43)	Caudal margin of male tergite nine produced into a pair of flattened black lobes, their medial edges coarsely toothed; outer gonostylus with tip curved and subacute, the margin of gonostylus with three or four teeth (see Alexander 1935b: Pl. II, fig. 31)	***Nephrotoma nigrostylata* Alexander, 1935** (China: Hubei, Zhejiang, Fujian, Sichuan, Guizhou, Guangxi)
–	Caudal margin of male tergite nine, and outer gonostylus not as above	**48**
47 (45)	Outer gonostylus abruptly narrowed at apical half; inner gonostylus with a relatively short beak (see Oosterbroek 1985: p. 250, figs. 36, 37)	***Nephrotoma relicta* (Savchenko, 1973)** (China: Heilongjiang, Sichuan, Hubei; Russia; North Korea; South Korea; Mongolia; Finland)
–	Outer gonostylus gradually narrowed; inner gonostylus with a relatively long beak (see Oosterbroek 1985: p. 250, figs 34, 35)	***Nephrotoma parvirostra* Alexander, 1924** (China: Beijing, Hebei, Heilongjiang, Hubei, Sichuan; Russia; Mongolia; South Korea; Japan)
48 (46)	Abdominal tergites without median stripe (see Alexander 1935b: p. 200)	***Nephrotoma evittata* Alexander, 1935** (China: Sichuan, Yunnan)
–	Abdominal tergites with median stripe	**49**
49 (48)	Male sternite eight with appendage in the middle of caudal margin (see Yang and Yang 1990: p. 478, Pl. III, fig. 1; Yang and Yang 1987b: p. 131, Pl. I, fig. 1A)	**50**
–	Male sternite eight without appendage in the middle of caudal margin (see Yang and Yang 1987a: p. 130, fig. 1B; Oosterbroek 1985: p. 271, fig. 111; Alexander 1935a: p. 135)	**51**
50 (49)	Male tergite nine with lobes acute apically (see Yang and Yang 1990: p. 478, Pl. III, fig. 2)	***Nephrotoma concava* Yang & Yang, 1990** (China: Gansu)
–	Male tergite nine with lobes rounded apically (see Yang and Yang 1987b: p. 131, Pl. I, fig. 1B)	***Nephrotoma hubeiensis* Yang & Yang, 1987** (China: Hubei)
51 (49)	Male tergite nine with two blunt processes on each side of hind margin (see Yang and Yang 1987a: p. 130, fig. 1C)	***Nephrotoma xizangensis* Yang & Yang, 1987** (China: Xizang)
–	Male tergite nine with two acute processes on each side of hind margin	**52**
52 (51)	Occipital mark brown, rounded; abdomen with segments seven to nine dark brown (see Alexander 1914: p. 162); male tergite nine with two spinous processes at caudal margin (see Oosterbroek 1985: p. 271, fig. 112)	***Nephrotoma repanda* (Alexander, 1914)** (China: Sichuan; Russia; North Korea; South Korea; Japan)
–	Occipital mark black, subtriangular; abdomen with all segments yellow (see Alexander 1935a: p. 134); male tergite nine with two horn-shaped processes at caudal margin (see Alexander 1935a: Pl. IV, fig. 43)	***Nephrotoma retenta* Alexander, 1935** (China: Sichuan-Xizang border)
53 (44)	Male tergite nine with process not directed caudally (see Tangelder 1984: p. 40, fig. 81)	***Nephrotoma libra* Alexander, 1951** (China: Xizang)
–	Male tergite nine with process directed caudally (see Yang and Yang 1987a: p. 131, fig. 2B; p. 132, fig. 3B)	**55**
54 (44)	Tergite nine dividing into four processes	**56**
–	Tergite nine dividing into two processes	**57**
55 (53)	Vertex with a light brown spot between eyes; male sternite nine with an appendage directed caudally in the middle of hind margin (see Yang and Yang 1987a: p. 132, fig. 3A)	***Nephrotoma didyma* Yang & Yang, 1987** (China: Xizang)
–	Vertex with a black spot between eyes; male sternite nine without appendage in the middle of hind margin (see Yang and Yang 1987a: p. 131, fig. 2A)	***Nephrotoma claviformis* Yang & Yang, 1987** (China: Xizang)
56 (54)	Inner gonostylus with crest	**58**
–	Inner gonostylus without crest	**59**
57 (54)	Male sternite eight with appendage in the middle of caudal margin	**67**
–	Male sternite eight without appendage in the middle of caudal margin	**68**
58 (56)	Inner gonostylus produced caudad into a long tail-like portion (see Alexander 1935a: Pl. IV, fig. 44, as *attenuata*); sternite eight without appendage in the middle of caudal margin (see Alexander 1935a: p. 136 as *attenuata*)	***Nephrotoma nigrohalterata* Edwards, 1928** (China: Sichuan, Xizang)
–	Inner gonostylus without such tail-like portion; sternite eight with an appendage in the middle of caudal margin	**60**
59 (56)	Male sternite eight with appendage in the middle of caudal margin (see Oosterbroek 1985: p. 244, fig. 10)	***Nephrotoma aculeata* (Loew, 1871)** (China: Heilongjiang, Shanxi; widely distributed in Palaearctic Region)
–	Male sternite eight without appendage in the middle of caudal margin	**62**
60 (58)	Occipital mark dark brown; male sternite nine with appendage in the middle of caudal margin (see Oosterbroek 1985: p. 272, fig. 115)	***Nephrotoma virgata* (Coquillett, 1898)** (China: Anhui, Hebei, Hubei, Sichuan, Zhejiang; Russia; North Korea; South Korea; Japan)
–	Occipital mark black; male sternite nine without appendage in the middle of caudal margin	**61**
61 (60)	Flagellum entirely brown; male sternite eight with a curved appendage directed ventrally at caudal margin (see Yang and Yang 1990: p. 124, fig. 3A)	***Nephrotoma ocellata* Yang & Yang, 1990** (China: Sichuan)
–	Flagellum entirely black; male sternite eight with a straight appendage directed caudad at caudal margin (see Oosterbroek 1984: p. 122, fig. 2)	***Nephrotoma cornicina cornicina* (Linnaeus, 1758)** (China: as far south as Zhejiang; widely distributed in Palaearctic Region)
62 (59)	Caudal margin of male tergite nine with two intermediate rounded processes and two spinous processes laterally (see Yang and Yang 1990: p. 124, fig. 2B)	***Nephrotoma kunagi* Yang & Yang, 1990** (China: Sichuan)
–	Caudal margin of male tergite nine with processes not as above	**63**
63 (62)	Cell m1 petiolate (see Alexander 1927: p. 182)	***Nephrotoma pleuromaculata* Alexander, 1927** (China: Hubei, Sichuan, Yunnan; India)
–	Cell m1 sessile	**64**
64 (63)	Intermediate lobes of male tergite nine with apex further produced into a pale triangular point (see Alexander 1935c: Pl. IV, fig. 43)	***Nephrotoma martynovi* Alexander, 1935** (China: Hebei, Heilongjiang, Inner Mongolia; Russia; Mongolia)
–	Intermediate lobes of male tergite nine with apex lacking such a point	**65**
65 (64)	Male tergite nine with a slender glabrous spine arising from the ventrolateral portion (see Alexander 1937: p. 25, fig. 29, as *brierei*)	***Nephrotoma scalaris parvinotata* (Brunetti, 1918)** (China: Beijing, Hebei, Shanxi, Shandong, Xinjiang, Inner mongolia, Gansu, Ningxia, Heilongjiang, Jiangsu, Anhui, Sichuan, Guizhou; Russia; Georgia; Armenia; Azerbaijan; Turkey; Syria; Lebanon; Iran; Kazakhstan; Turkmenistan; Uzbekistan; Tajikistan; Kyrgyzstan; Afghanistan; Mongolia; India; Pakistan)
–	Male tergite nine without such a spine arising from the ventrolateral portion (see Oosterbroek 1985: p. 259, fig. 72)	***Nephrotoma pullata* (Alexander, 1914)** (China: Heilongjiang; Russia; North Korea; Japan)
66 (29)	Male sternite eight with a small compressed lobe at posterior margin (see Alexander 1935a: p. 139)	***Nephrotoma pilata* Alexander, 1935** (China: Sichuan)
–	Male sternite eight lacking such a lobe at posterior margin (see Alexander 1949: p. 519)	***Nephrotoma vesta* Alexander, 1949** (China: Guangdong)
67 (57)	Abdominal tergites black with pale yellow lateral stripes	**69**
–	Abdominal tergites yellow with dark lateral stripes	**70**
68 (57)	Inner gonostylus without crest	**71**
–	Inner gonostylus with crest	**72**
69 (67)	Antennae entirely black; male tergite nine terminating in two rounded lobes at caudal margin (see Alexander 1935a: Pl. IV, fig. 49)	***Nephrotoma biformis* Alexander, 1935** (China: Sichuan)
–	Antennae entirely brown; male tergite nine terminating in two acute lobes at caudal margin (see Yang and Yang 1990: p. 481, Pl. V, fig. 2)	***Nephrotoma joneensis* Yang & Yang, 1990** (China: Gansu)
70 (67)	Cell m1 petiolate (see Yang and Yang 1987: p. 245)	***Nephrotoma barbigera* (Savchenko, 1964)** (China: Heilongjiang, Jilin, Gansu, Ningxia, Shanxi; Russia)
–	Cell m1 sessile	**77**
71 (68)	Male tergite nine with a pair of spinous processes except setae	**73**
–	Male tergite nine without spinous processes except setae (see Oosterbroek 1985: p. 262, fig. 79)	***Nephrotoma bifusca* Alexander, 1920** (China: Heilongjiang, Hebei, Jilin; Russia; North Korea; South Korea; Japan)
72 (68)	Cell m1 petiolate	**75**
–	Cell m1 sessile	**76**
73 (71)	Male tergite nine projected into a pair of spike-like processes at caudal margin, each with a series of six or seven blackened points along their medial edge (see Savchenko 1973: p. 47)	**74**
–	Male tergite nine projected into a pair of fingerlike processes at caudal margin, the horn without points described as above (see Yang and Yang 1990: p. 480, Pl. IV, fig. 2)	***Nephrotoma shanxiensis* Yang & Yang, 1990** (China: Shanxi)
74 (73)	Cell m1 petiolate (see Alexander 1935a: Pl. I, fig. 19)	***Nephrotoma impigra impigra* Alexander, 1935** (China: Zhejiang, Hubei, Jiangxi, Fujian, Sichuan)
–	Cell m1 sessile (see Savchenko 1973: p. 48)	***Nephrotoma impigra fulvovittata* (Savchenko, 1964)** (China: Yunnan)
75 (72)	Male tergite nine terminating in two angular lobes at caudal margin (see Tangelder 1984: p. 74, fig. 235)	***Nephrotoma quadristriata* (Schummel, 1833)** (China: Xinjiang; widely distributed in Palaearctic Region)
–	Male tergite nine terminating in two truncated lobes at caudal margin (see Tangelder 1984: p. 49, fig. 115)	***Nephrotoma spicula* Tangelder, 1984** (China: Heilongjiang; Russia; North Korea)
76 (72)	Inner gonostylus with crest toothed (see Tangelder 1984: p. 77, fig. 245)	***Nephrotoma scurra* (Meigen, 1818)** (China: Gansu, Inner Mongolia; widely distributed in Palaearctic Region)
–	Inner gonostylus with crest not toothed (see Tangelder 1984: p. 28, fig. 20; p. 30, fig. 29; p. 38, fig. 73)	**78**
77 (70)	Male tergite nine terminating in two truncated lobes at caudal margin; inner gonostylus with an angular process on dorsal side (see Mannheims 1967: p. 178, fig. 1)	***Nephrotoma ligulata* Alexander, 1925** (China: Xinjiang; Russia; Turkmenistan; Uzbekistan; Tajikistan; Kyrgyzstan; Afghanistan; Mongolia; India)
–	Male tergite nine terminating in two bluntly rounded lobes at caudal margin; inner gonostylus without process on dorsal side (see Yang and Yang 1987: p. 244, figs. 3B, 3C)	***Nephrotoma xinjiangensis*** Yang & Yang, 1987 (China: Xinjiang)
78 (76)	Male sternite nine with an appendage directed caudally (see Alexander 1953: p. 332, fig. 3a)	***Nephrotoma kaulbacki*** Alexander, 1951 (China: Xizang)
–	Male sternite nine with an appendage directed ventrally (see Tangelder 1984: p. 28, fig. 16; p. 30, fig. 27)	**79**
79 (78)	Abdominal tergites without lateral stripes; male tergite nine separated by a U-shaped notch medially on caudal margin (see Tangelder 1984: p. 30, fig. 30)	***Nephrotoma perobliqua*** Alexander, 1936 (China: Gansu)
–	Abdominal tergites with lateral stripes; male tergite nine separated by a V-shaped notch medially on caudal margin (see Tangelder 1984: p. 28, fig. 18)	***Nephrotoma pjotri*** Tangelder, 1984 (China: Xinjiang; Kazakhstan; Uzbekistan; Kyrgyzstan)

## Taxonomy

### Nephrotoma liankangensis sp. n.

Taxon classificationAnimaliaDipteraTipulidae

http://zoobank.org/50E64607-C1E1-4AD1-88AB-10F98DF3FF37

Figs 1–9 , 10–19

#### Diagnosis.

Antennae with flagellum light brown, pleura light yellow conspicuously patterned with white, abdominal tergites with two black lateral stripes and one brown median stripe, ninth tergite with two rounded lobes which are densely covered with black spines, posterior margin of ninth tergite not concaved at base of lobes.

#### Description.

Male (n=2): body length 9.8 mm, wing 10.5 mm, antenna 5.8 mm.

**Figures 1–9.**
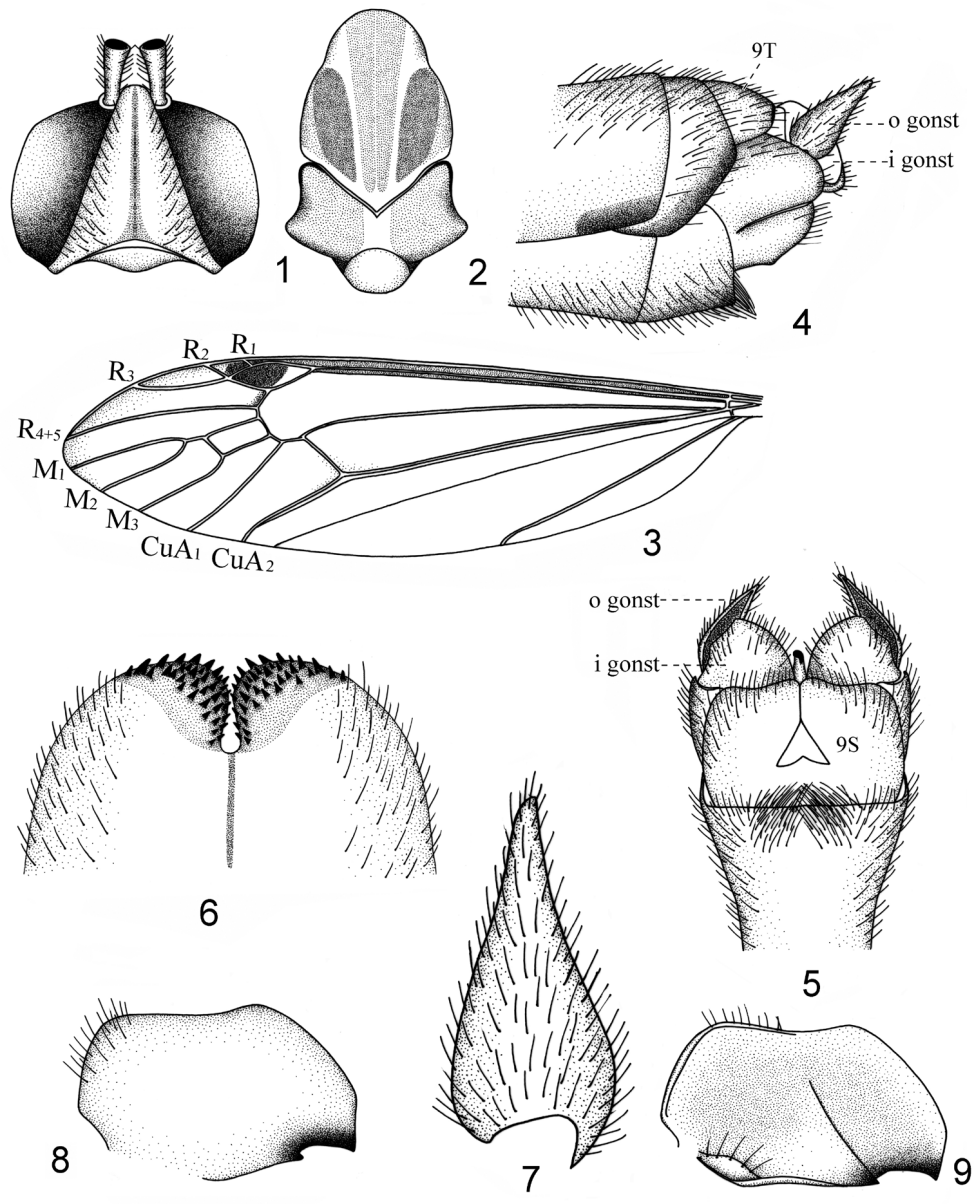
*Nephrotoma liankangensis* sp. n. **1** head, dorsal view **2** thorax, dorsal view **3** left wing **4** hypopygium, lateral view **5** hypopygium, ventral view **6** ninth tergite, dorsal view **7** outer gonostylus, lateral view **8** inner gonostylus, lateral internal view **9** inner gonostylus, lateral external view. Abbreviations: i gonst = inner gonostylus, o gonst = outer gonostylus, S = stergite.

*Nasus* yellow with black setae, palpi black. Antennae relatively long, if bent backward extending to the first abdominal tergite, scape and pedicel yellow, flagellum 10-segmented, with the first flagellomere yellow, with the second to tenth flagellomeres light brown and enlarged at both ends, the basal enlargement black with black setae basally, subequal to the flagellomeres from which they arise. Head yellow, occipital brand brown and narrow, along the middle line of occiput (Fig. 1).

*Pronotum* light brown, changing into yellow in middle. Prescutum yellow with three brown stripes, median one expanded apically, not extending to the hind border, lateral stripe straight and rounded apically, extending slight beyond the middle of median stripe (Fig. 2). The median stripe lighter in coloration than lateral one, sometimes divided by a yellow line (Fig. 2). Scutum light brown, each lobe with jet-black anterior border, median area of scutum yellow (Fig. 2). Scutellum yellowish. Postnotum light brown. Pleura yellowish brown, variegated by white on anepimeron, katepimeron, meron and basal laterotergite. Halters yellowish brown throughout. Legs with coxae and trochanters yellow; femora and tibiae yellow with brown tips; tarsi dark brown. Wings transparent, cells c and sc suffused with brown; stigma oval, dark brown; wing tip narrowed and slightly suffused with light brown. Cell r1 without stigmal trichia, cell m1 petiolate (Fig. 3).

*Abdomen* generally yellow, the first tergite light brown, tergites two to six with a light brown median stripe and two black lateral stripes, sternites two to seven with light brown median stripe, hypopygium chiefly yellowish brown. Male hypopygium (Figs 4, 5) with the ninth tergite having the median notch rounded basally, gradually narrowed to apex, separating the ninth tergite into two rounded lobes, the lobe black and densely covered with black spines (Fig. 6). Outer gonostylus lanceolate, basally widened and gradually narrowed to the end (Figs 4, 5, 7). Inner gonostylus with two black beaks (Figs 8, 9).

**Figures 10–19.**
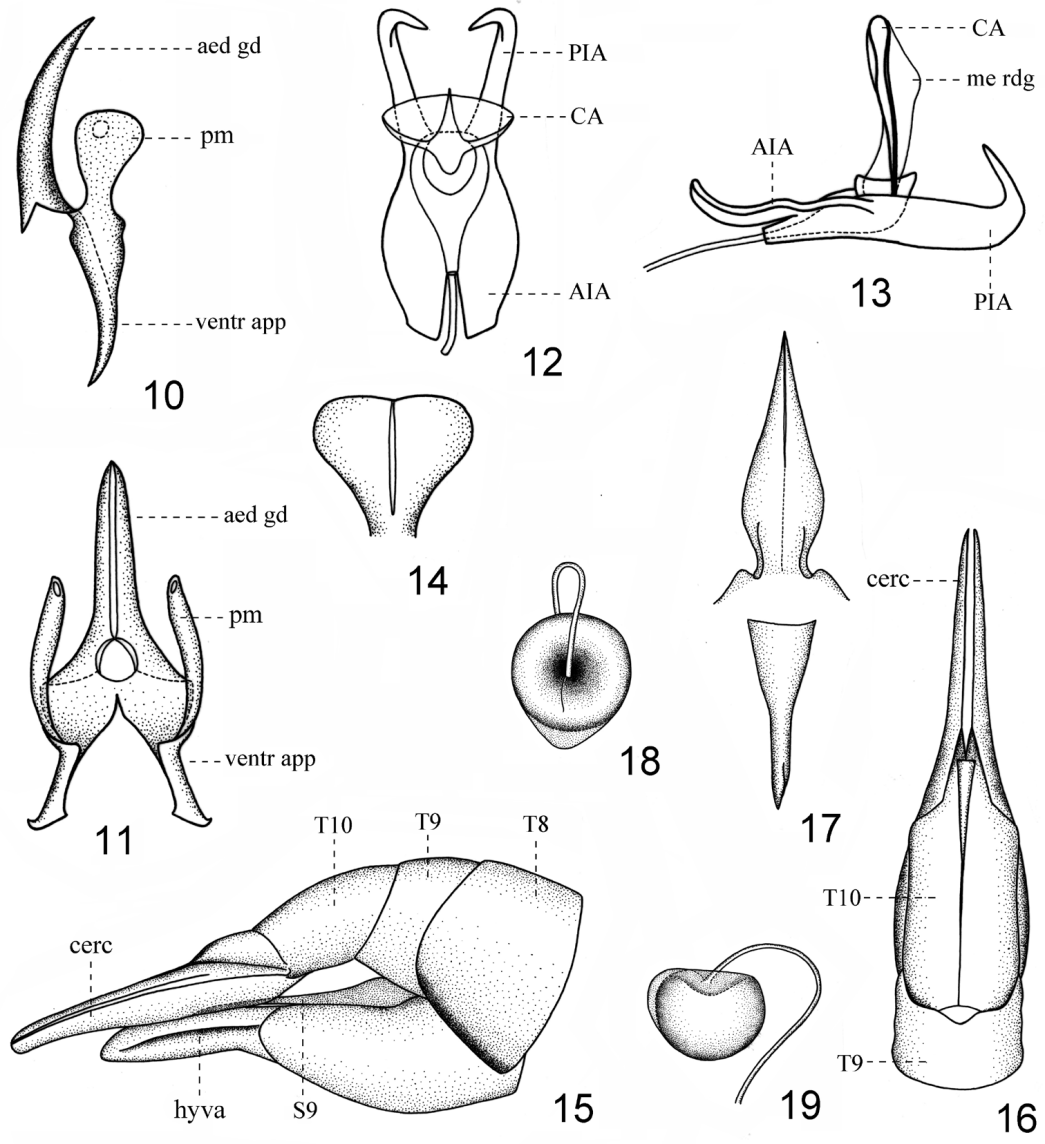
*Nephrotoma liankangensis* sp. n. **10** aedeagal guide, lateral view **11** aedeagal guide, dorsal view **12** semen pump, dorsal view **13** semen pump, lateral view **14** compressor apodeme **15** ovipositor, lateral view **16** ovipositor, dorsal view **17** ninth stergite and vaginal apodeme, dorsal view **18** spermatheca, dorsal view **19** spermatheca, lateral view. Abbreviations: aed gd = aedeagal guide, pm = paramere, ventr app = ventral appendage of aedeagal guide, PIA = posterior immovable apodeme, AIA = anterior immovable apodeme, CA = compressor apodeme, me rdg = median ridge, T = tergite, cerc = cercus, hyva = hypovalva.

*Aedeagal guide* horn-shaped in lateral view, very acute apically (Fig. 10); paramere lamellate, blunt apically (Fig. 10); ventral appendage of aedeagal guide horn-like in lateral view, boot-shaped in dorsal view (Figs 10, 11).

*Semen pump* with posterior immovable apodeme (PIA) narrow, dorsally bent in lateral view (Fig. 13); compressor apodeme (CA) fan-shaped, with an obviously expanded median ridge in lateral view, in a 90° angle with posterior immovable apodeme (Figs 12–14); anterior immovable apodeme (AIA) broader than compressor apodeme, with lateral margins arched and inner margins straight, separated medially (Fig. 12).

Female (n=2): body length 16.2 mm, wing 12.0 mm, antenna 3.2 mm.

The general coloration of head, thorax and abdomen similar to that of male.

*Antennae* relatively short, if bent backward not extending to abdomen, scape and pedicel yellow, flagellum 10-segmented, each flagellomere cylindrical, gradually shorter and slightly enlarged at base, the basal two flagellomeres yellow, three to ten flagellomeres yellowish brown with black at base.

*Ovipositor* yellowish brown, ninth sternite very thin, lanceolate, separated medially (Fig. 17). Sternite ten slightly shorter than cerci, parallel in lateral margins in dorsal view (Fig. 16). Cerci long, acinacifoliate, widened at basal three fifths, narrowed at apical two fifths, surpassing the end of hypovalva (Fig. 15). Hypovalva simple, extending to nearly three quarters length of cerci (Fig. 15).

*Vaginal apodeme* widened at basal half and gradually tapered to the end, very acute apically (Fig. 17). Spermatheca spherical, brown, well-sclerotized, with membranous angular extension on lateral side (Figs 18, 19).

#### Material examined.

**Holotype** male. Pinned specimen. **China**: Henan Province, Xin County, Liankangshan National Nature Reserve, 18 Jul. 2014, coll. Qiulei Men. **Paratype.** Pinned specimen. **China**: 1 male 2 females, same data as holotype, coll. Wu Zeng.

#### Distribution.

China (Henan).

#### Etymology.

The specific epithet is a noun ‘*liankang*’ with Latin suffix ‘*ensis*’, referring to the distribution of the new species.

#### Remarks.

This new species is similar to *Nephrotoma pseudoliankangensis*, as discussed below, and another Chinese species, *Nephrotoma sinensis* (Edwards, 1916), by the coloration of the prescutum and wings, the shape of lobes on the ninth tergite and the shape of inner gonostylus. It can be easily distinguished from the latter by the prescutum not bearing a black spot on anterior portion of the lateral stripe (this black spot present in *Nephrotoma sinensis* as described by Liu et al. 2009: 43), the ninth tergite without spiny prominence on each side of lobes (this spiny prominence on each side of lobes in *Nephrotoma sinensis* as illustrated by Sidorenko 1999: 112), and the inner gonostylus broad apically (apical half obviously narrower than that of *Nephrotoma liankangensis* in *Nephrotoma sinensis* as illustrated by Sidorenko 1999: 112).

### Nephrotoma pseudoliankangensis sp. n.

Taxon classificationAnimaliaDipteraTipulidae

http://zoobank.org/5AAE0CD7-E0D8-454E-9EC0-173267C91215

Figs 20–27 , 28–36

#### Diagnosis.

General coloration light yellow, antennae with flagellum black except the first flagellomere, pleura white conspicuously patterned with yellow, abdominal tergites with two black lateral stripes and one brown median stripe, ninth tergite with two rounded lobes, posterior margin of ninth tergite slightly concaved at base of lobes.

#### Description.

Male (n=2): body length 9.6 mm, wing 11.0 mm, antenna 5.1 mm.

**Figures 20–27.**
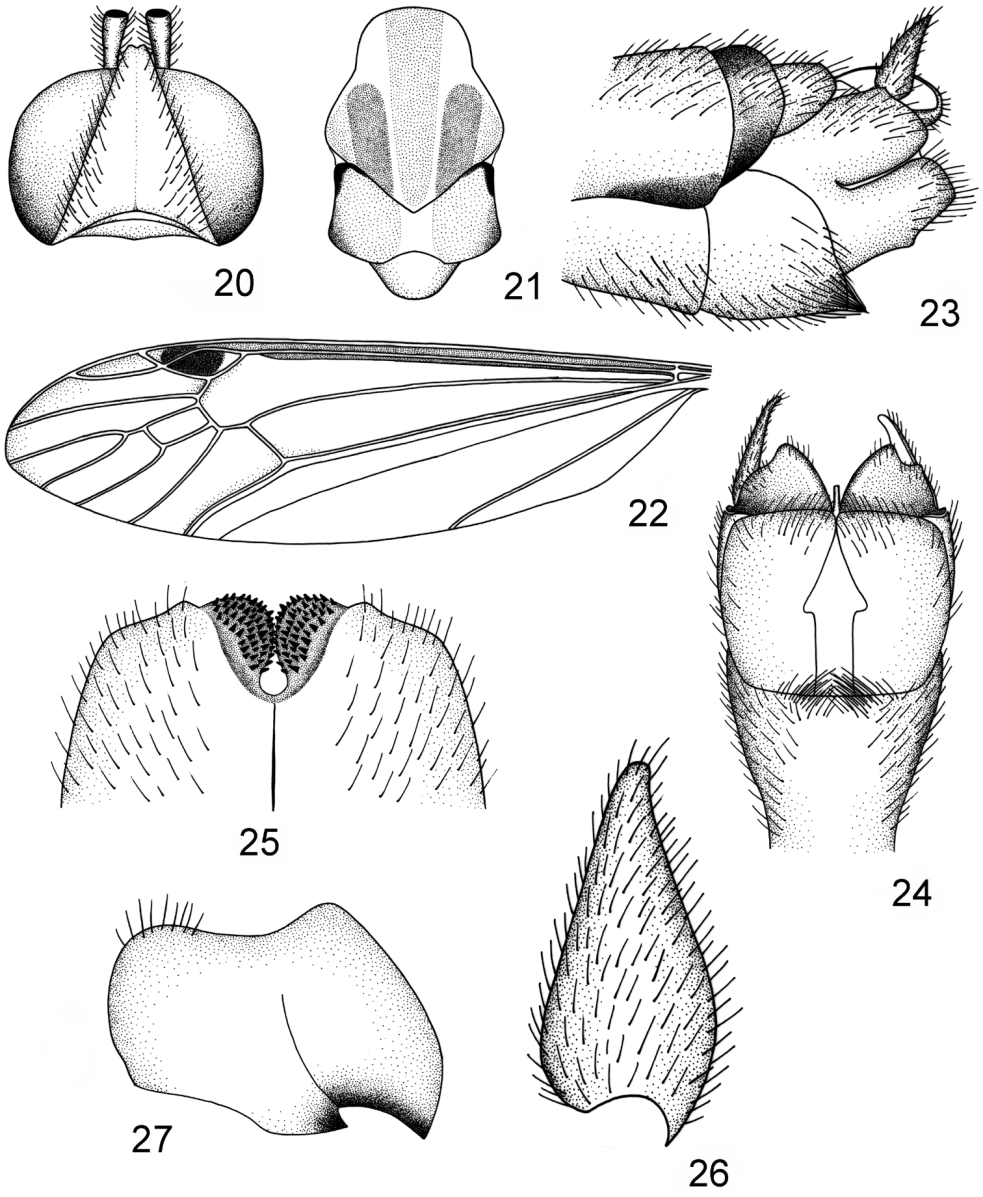
*Nephrotoma pseudoliankangensis* sp. n. **20** head, dorsal view **21** thorax, dorsal view **22** left wing **23** hypopygium, lateral view **24** hypopygium, ventral view **25** ninth tergite, dorsal view **26** outer gonostylus, lateral view **27** inner gonostylus, lateral view.

*Nasus* brown with brown setae, palpi black. Antennae relatively long, if bent backward extending to the first abdominal tergite, scape and pedicel yellow, flagellum 10-segmented, with the first flagellomere yellow, with second to tenth flagellomeres black and enlarged at base and apex, each flagellomere with black setae at base, subequal to the flagellomeres where they are found. Head yellow without occipital brand (Fig. 20).

*Pronotum* entirely yellow. Prescutum yellow with three brown stripes, median one percurrent and expanded apically, lateral stripe straight and rounded apically, the area between lateral stripe and lateral border of prescutum suffused with brown (Fig. 21). Scutum light brown, each lobe with jet-black anterior border, median area of scutum yellow (Fig. 21). Scutellum yellowish brown. Postnotum light brown with white median stripe. Pleura white, variegated by yellow on anepisternum and katepisternum. Halters yellowish brown throughout. Legs with coxae and trochanters yellow; femora and tibiae yellow with brown tips; tarsi dark brown. Wings transparent, cells c and sc variegated with brown; stigma oval, dark brown; wing tip narrowed and slightly suffused with light brown. Cell r1 with five to six stigmal trichiae, cell m1 petiolate (Fig. 22).

*Abdomen* generally yellow, the first segment yellow with light brown tergite, tergites two to seven with a brown median stripe and two black lateral stripes, the median stripe expanded at hind border, tergite eight entirely brownish black; sternites two to seven with light brown median stripe, hypopygium chiefly yellowish brown. Male hypopygium (Figs 23, 24) with the ninth tergite having the median notch rounded and widened basally, separating the ninth tergite into two rounded black lobes, which densely covered with black spines, almost connected to each other (Fig. 25). Outer gonostylus lanceolate, basally widened and gradually narrowed to the end (Figs 23, 24, 26). Inner gonostylus with two black beaks, the dorsal side of inner gonostylus obviously extended (Fig. 27).

**Figures 28–36.**
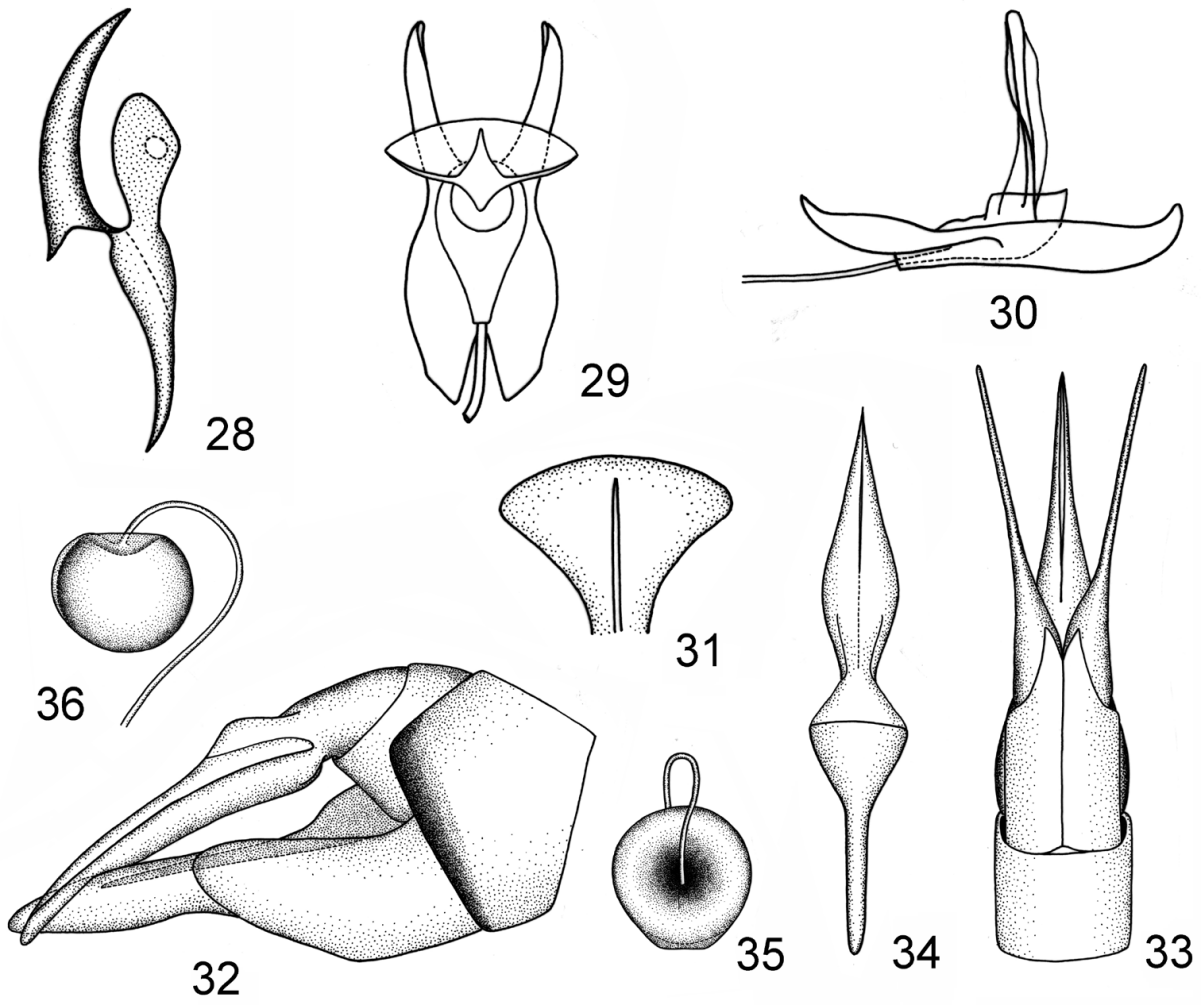
*Nephrotoma pseudoliankangensis* sp. n. **28** aedeagal guide, lateral view **29** semen pump, dorsal view **30** semen pump, lateral view **31** compressor apodeme **32** ovipositor, lateral view **33** ovipositor, dorsal view **34** ninth stergite and vaginal apodeme, dorsal view **35** spermatheca, dorsal view **36** spermatheca, lateral view.

*Aedeagal guide* very similar to that of *Nephrotoma liankangensis*, with paramere not blunt apically, the dorsal margin slightly arched, the ventral and outer margins forming an obtuse angle (Fig. 28).

*Semen pump* very similar to that of *Nephrotoma liankangensis*, with posterior immovable apodeme (PIA) not bent directed dorsad at the apex in lateral view (Fig. 30); compressor apodeme (CA) fan-shaped, broader than that of *Nephrotoma liankangensis* (Figs 29, 31), with median ridge slightly expanded in lateral view, which more degenerated than that of *Nephrotoma liankangensis* (Fig. 30); anterior immovable apodeme (AIA) more expanded than that of *Nephrotoma liankangensis* in lateral view (Fig. 30).

Female (n=3): body length 15.8 mm, wing 13.0 mm, antenna 2.5 mm.

The colouration of head, thorax and abdomen similar to those of male.

*Antennae* relatively short, scape and pedicel yellow, flagellum 10-segmented, each flagellomere cylindrical, gradually shorter, the basal two flagellomeres entirely yellow, three to ten flagellomeres yellowish brown with black at base.

*Ovipositor* (Figs 32, 33) very similar to that of *Nephrotoma liankangensis*, with ninth sternite obviously longer than that of *Nephrotoma liankangensis* (Fig. 34).

*Vaginal apodeme* widened at basal two fifths, the rest of vaginal apodeme tubular, parallel, acute apically (Fig. 34).

*Spermatheca* spherical, brown, well-sclerotized, with membranous extension truncate (Figs 35, 36).

#### Material examined.

**Holotype** male. Pinned specimen. **China**: Yunnan Province, Kunming, Baofeng wetland park, 31 Aug. 2013, coll. Bin Zhang. **Paratype.** Pinned specimen. **China**: 1 male 3 females, same data as holotype.

#### Distribution.

China (Yunnan).

#### Etymology.

The specific epithet is based on the name of the related species, *Nephrotoma liankangensis*, with the Latin prefix ‘*pseudo*’, referring to the morphological similarity of the new species to *Nephrotoma liankangensis*.

#### Remarks.

This new species is externally similar to *Nephrotoma liankangensis* by the colouration of head, thorax, abdomen and wings, and the shape of inner and outer gonostyli. It can be easily distinguished from the latter by antennae with the second to tenth flagellomeres black (the second to tenth flagellomeres yellow with basal enlargement black in *Nephrotoma liankangensis*), the occiput unpatterned (with brown median brand in *Nephrotoma liankangensis* as shown in Fig. 20), and the inner gonostylus with the dorsal margin obviously extended (just slightly extended in *Nephrotoma liankangensis* as shown in Figs 8, 9). The new species is also similar to *Nephrotoma liankangensis* in some internal reproductive organs, but differs from the latter in the female vaginal apodeme widened at basal two fifths, tubular and parallel at apical three fifths (vaginal apodeme widened at basal half and gradually tapered to the end in *Nephrotoma liankangensis* as shown in Fig. 17), the spermatheca laterally with membranous extension truncate (with membranous extension angular in *Nephrotoma liankangensis* as shown in Fig. 18), the male aedeagal guide with paramere not blunt apically (apically blunt in *Nephrotoma liankangensis* as shown in Fig. 10), the semen pump with posterior immovable apodeme not dorsally bent (dorsally bent in *Nephrotoma liankangensis* as shown in Fig. 13), and compressor apodeme with median ridge slightly expanded in lateral view (median ridge obviously more expanded than that of *Nephrotoma pseudoliankangensis* in *Nephrotoma liankangensis* as shown in Fig. 13).

## Discussion

Based on morphological comparison, the characters of the vaginal apodeme, spermatheca and semen pump are uniform in different individuals of *Nephrotoma pseudoliankangensis* and *Nephrotoma liankangensis*. However, these structures show noticeable differences between the two species. More comparative morphological study may not only prove a high application value of the characters of these internal reproductive structures in separating relative species, but also prove the roles of these structures for phylogenetic studies as mentioned by Tangelder (1985). Moreover, although used less frequently, the characteristics of female genitalia, especially the vaginal apodeme and ninth sternite, may be helpful to effectively distinguish species in which males are difficult to collect.

## Supplementary Material

XML Treatment for Nephrotoma liankangensis

XML Treatment for Nephrotoma pseudoliankangensis
